# Draft Genome Sequence of *Gluconobacter oxydans* NL71, a Strain That Efficiently Biocatalyzes Xylose to Xylonic Acid at a High Concentration

**DOI:** 10.1128/genomeA.00615-15

**Published:** 2015-06-18

**Authors:** Yuanyuan Miao, Xin Zhou, Yong Xu, Shiyuan Yu

**Affiliations:** a College of Chemical Engineering, Nanjing Forestry University, Nanjing, China; b Jiangsu Province Key Laboratory of Biomass-Based Green Fuels and Chemicals, Nanjing, China; c Key Laboratory of Forest Genetics and Biotechnology, MED, Nanjing, China

## Abstract

*Gluconobacter oxydans* NL71, a selected strain in the crude lignocellulosic hydrolysate, catalyzed 600 g/liter xylose to 586.3 g/liter xylonic acid at 95.1% yield. The biocatalysis of xylose yielded three times higher than the best previous output, providing a possibility of the industrial scale utilization of lignocellulosic xylose. Due to its promising industrial applications, we sequenced the complete genome of strain *G. oxydans* NL71 to further our understanding of its overall metabolism.

## GENOME ANNOUNCEMENT

*Gluconobacter oxydans*, a Gram-negative and obligate aerobe of the genus *Acetobacteraceae*, has the ability to incompletely oxidize sugars, alcohols, and aldehydes, leading to the accumulation of organic acids as end products. The high yields of corresponding products are secreted almost completely into the medium, even in highly concentrated sugar and strong acidic solutions, which makes *G. oxydans* important for industrial production (1). Through culture screening of the crude lignocellulosic hydrolysate, which was prepared by a dilute sulfuric acid steam-exploded corn stover, we obtained *Gluconobacter oxydans* NL71 from *Gluconobacter oxydans* 621H. *G. oxydans* NL71 produced 586.3 g/liter of xylonic acid efficiently from 600 g/liter of xylose, at 95.1% yield and 4.69 g/liter/h volumetric productivity. Presently, this level is the highest observed for xylose bioconversion. Furthermore, *G. oxydans* NL71 directly produced 143.9 g/liter of xylonic acid from the diluted sulfuric acid prehydrolysates of corn stover without any detoxification step, at 96.9% yield and 1.0 g/liter/h volumetric productivity (2). Here, we present the draft genome of *G. oxydans* NL71. The elucidation of the genome sequence might provide a basis for both evolutionary analysis and improvement of the biotechnological applications of the organism.

The genome of *G. oxydans* NL71 was sequenced at the Novogene Bioinformatics Institute (Beijing, China) with MPS (massively parallel sequencing) Illumina technology. Draft assemblies were based on 342-Mb (Illumina MiSeq) and 758-Mb (Illumina HiSeq 2500) total reads. The reads provided 100-fold coverage and 222-fold coverage of the genome, respectively. The reads were assembled by Short Oligonucleotide Alignment Program (SOAP) *de novo* software (3, 4) into 6 scaffolds and 12 contigs. Physical gaps, repeats, and assembly ambiguities were corrected by multiplex PCR and Sanger sequencing, and tRNAs and rRNAs were predicted with tRNAscan-SE (5) and RNAmmer (6). Gene prediction was performed on the *G. oxydans* NL71 genome assembly by GeneMarkS (7) with an integrated model that combined the GeneMarkS generated (native) and heuristic model parameters. A whole-genome BLAST (8) search was performed against 6 databases: KEGG, COG, NR, Swiss-Prot, GO, and TrEMBL (9–12).

The total size of the complete genome of *G. oxydans* NL71 is 3,403,780 bp with a G+C content of 55.71%, which is similar to other *Gluconobacter*. The draft genome annotation revealed 3,226 predicted protein-coding sequences (CDSs). There are a total of 5 copies of rRNA operons and 59 genes encoding tRNAs found on the chromosome. The genome sequences of *G. oxydans* 621H (2.7 Mbp) (GenBank accession numbers CP000004 to CP000009) were used as references (13). In general, on the basis of the genomic sequence of *G. oxydans* NL71, we hope to conduct further studies to try to understand in more detail the possible metabolic mechanisms for its great biotransformation of d-xylose into d-xylonate, especially the cellular resistance to highly concentrated xylose and xylonate solution, and various inhibitors in the crude lignocellulosic hydrolyzates (14). Furthermore, the genome sequence will be important to improve the industrial-scale production of d-xylonate.

### Nucleotide sequence accession numbers. 

This whole-genome shotgun project has been deposited at DDBJ/EMBL/GenBank under the accession number LCTG00000000. The version described in this paper is version LCTG00000000.1.
